# In Vitro Effects of Lemon Balm Extracts in Reducing the Growth and Mycotoxins Biosynthesis of *Fusarium culmorum* and *F. proliferatum*

**DOI:** 10.3390/toxins14050355

**Published:** 2022-05-19

**Authors:** Pascaline Aimee Uwineza, Monika Urbaniak, Marcin Bryła, Łukasz Stępień, Marta Modrzewska, Agnieszka Waśkiewicz

**Affiliations:** 1 Department of Chemistry, Poznań University of Life Sciences, Wojska Polskiego 75, 60-625 Poznań, Poland; agat@up.poznan.pl; 2 Pathogen Genetics and Plant Resistance Department, Institute of Plant Genetics, Polish Academy of Sciences, 60-479 Poznań, Poland; murb@igr.poznan.pl; 3 Department of Food Safety and Chemical Analysis, Prof. Waclaw Dabrowski Institute of Agricultural and Food Biotechnology—State Research Institute, Rakowiecka 36, 02-532 Warsaw, Poland; marcin.bryla@ibprs.pl (M.B.); marta.modrzewska@ibprs.pl (M.M.); 4 Department of Plant-Pathogen Interaction, Institute of Plant Genetics, Polish Academy of Sciences, 60-479 Poznań, Poland; lste@igr.poznan.pl

**Keywords:** plant extracts, modified mycotoxins, *Fusarium*, UHPLC-HESI-MS/MS, ergosterol

## Abstract

The objectives of this research were to obtain the extracts of lemon balm (*Melissa officinalis*) using supercritical CO_2_ (SC-CO_2_) and methanol as co-solvent and evaluate the antifungal activity of those extracts against two selected strains of *Fusarium* species (*Fusarium culmorum* and *Fusarium proliferatum*). The extraction conditions were set at 40 and 60 °C and 250 bar. The obtained extracts were characterized in terms of antifungal activity on potato dextrose agar media (PDA). The results showed that the extraction parameters had different effects on mycelium growth and mycotoxins biosynthesis reduction. All studied lemon balm extracts (1, 2.5, 5, 7.5, and 10%) inhibited the growth of *F. proliferatum* and *F. culmorum* mycelia compared to the control. The lemon balm extracts significantly reduced ergosterol content and synthesized mycotoxins in both tested strains. These findings support the antifungal activity of lemon balm extracts against *F. proliferatum* and *F. culmorum*. However, more research on other *Fusarium* species is needed, as well as in vivo applications, before considering lemon balm extracts as a natural alternative to synthetic fungicides.

## 1. Introduction

Fungal infections are among the world’s most destructive agricultural diseases. Various species of the *Fusarium* genus, particularly *Fusarium culmorum* and *Fusarium proliferatum,* are naturally prevalent and globally cause the qualitative and quantitative losses of farming commodities, mainly cereals, cereal-based products, fruit, and vegetables [1,2,3,4]. Based on available literature data, crop yield reductions ranging from 10 to 40% have been reported [5,6,7]. Along with their pathogenicity to various plants, these *Fusarium* spp. can synthesize secondary metabolites known as mycotoxins under favorable conditions–high temperature, humidity, and moisture in the field before harvest, post-harvest, during processing, or in storage [2,7,8]. Furthermore, most mycotoxins produced by *Fusarium* spp. are heat-stable. Some cause serious health concerns in humans and farm animals, including mutagenic, teratogenic, neurotoxic, carcinogenic, and estrogenic effects [9,10,11]. However, mycotoxin’s action is determined by the toxin type and its concentration in the contaminated food or feed.

*Fusarium culmorum* is one of the most important pathogens of cereals, most notably wheat and barley. It is most prevalent in areas with mild to cold temperatures, and it is primarily responsible for the Fusarium head blight *(*FHB*)* as well as foot and root rot (FRR) diseases [12]. Moreover, *F. culmorum* produces a variety of mycotoxins, including type B trichothecenes (nivalenol–NIV and deoxynivalenol–DON), as well as zearalenone (ZEN) and fusarenon-X. These mycotoxins (NIV and DON) interrupt cell functions by attaching to the ribosome and preventing protein synthesis [12]. 

On the other hand, *F. proliferatum* is a pathogenic fungus that primarily contaminates maize. It is known as a common field pathogen but can also be found in storage when the moisture level is high, and the temperature is low [13,14]; for example, it was reported in stored garlic [15]. *F. proliferatum* is an essential producer of several mycotoxins, particularly fumonisin B_1_ [16], regarded as a group B carcinogen in humans [17]. Additionally, fumonisin B_1_ has been implicated in esophageal and liver cancers [18,19,20] and neural tube defects [21].

Fungal infections and mycotoxins’ biosynthesis in various crop varieties are a big concern worldwide. Thus, many farmers and food producers have adopted synthetic fungicides on fields or in stored products as one of the most successful and cost-effective ways to combat toxigenic fungi and contamination with their mycotoxins [22]. However, due to the widespread use of synthetic fungicides, different side effects have been noticed, such as the development of resistance among the target microorganisms, toxicity to humans, animals, and other non-target organisms, and environmental contamination due to residual toxicities [23]. Therefore, several researchers and institutions have focused on developing alternatives to synthetic fungicides for management strategies of mycotoxigenic fungi, including biocontrol agents, the selection of resistant cultivars, and agronomic approaches (e.g., soil tillage and crop rotation) [24,25].

Among others, biocontrol agents, specifically bioactive metabolites such as essential oils and plant extracts, have gained popularity nowadays as alternatives to synthetic fungicides not only in terms of sustainable development and ecological protection of nature, wildlife, and agriculture commodities [26]. Apart from that, it is an innovative approach considered environmentally safe, biodegradable, effective, and economically practical [13,27,28,29,30,31,32]. Lemon balm (*Melissa officinalis*) is a fragrant and medicinal herb belonging to the *Lamiaceae* family. This plant grows natively in many world regions, including Iran, Southern Europe, Northern Africa, and Central Asia [33,34]. For more than 2000 years, various communities have used this plant for different purposes, such as regulating sleep, appetite, digestion, reducing anxiety, and pain relief [35,36]. Today, it is one of the most widely utilized medicinal plants in various sectors, particularly medicines, cosmetics, nutraceuticals, and food preservation. Lemon balm contains diverse compounds, including tannins, volatile compounds (e.g., neral, geranial, citronellal, and geraniol), phenolic acids (ferulic, hydroxycinnamic, caffeic acid, caffeic acid derivatives, especially rosmarinic acid), terpenes (triterpenes, monoterpenes, and sesquiterpenes) and flavonoids [37]. Lemon balm contains low amounts of essential oils 0.02–0.30%, mostly monoterpenes, such as citronellal, neral, or geranial [38]. Moreover, the antioxidative and antimicrobial effects of lemon balm essential oils and extracts have been reported [39,40,41,42,43,44]. 

Many extraction techniques have been developed to extract lemon balm, including steam distillation, hydro-distillation, solvent extraction (methanol, ethanol), aqueous extraction, and microwave extraction [45,46,47,48,49]. Supercritical fluid extraction (SFE) is one of the most efficient and eco-friendly techniques for extracting secondary metabolites from plants that uses the characteristics of fluids at their critical points to extract soluble components from a variety of biological matrices [38,50]. In the supercritical state, solvents have the properties of both liquids and gases, such as low viscosity, high diffusivity, and density, which are beneficial in extractive activities [51]. CO_2_ is a popular solvent for SFE because it is readily available, non-toxic, non-flammable, cost-effective, and easy to remove from the final extract while keeping its biological properties [52]. Although many reports are available on lemon balm extraction and its efficacy as an antimicrobial agent, very few used supercritical CO_2_ as an extraction solvent. Further investigation is required to understand the inhibitory effect of lemon balm extracts on phytopathogenic fungi, particularly *Fusarium* species and their mycotoxins. Therefore, the aims of this research were: (i) extraction of lemon balm by supercritical CO_2_ under various conditions, (ii) evaluation of antifungal efficacy of lemon balm extracts against two strains of *Fusarium* species, and (iii) quantitative determination of ergosterol (ERG) and produced mycotoxins.

## 2. Results

### 2.1. The Inhibitory Effect of Lemon Balm Extracts on Fusarium Growth 

**Figure 1:**
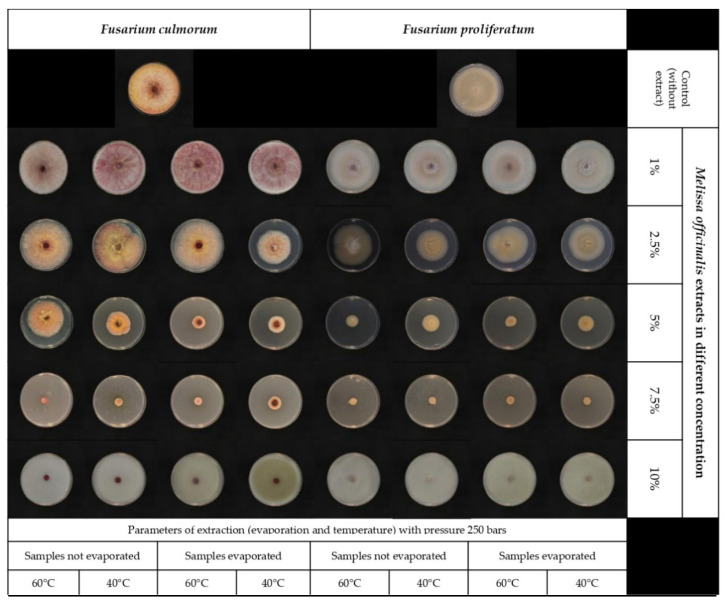
The inhibitory effects of lemon balm extracts at different concentration levels (1–10%) on mycelia growth of *F. culmorum* and *F. proliferatum* in PDA medium.

This study looked at the antifungal activity of lemon balm extracts produced using supercritical CO_2_ extraction under various parameters. The effectiveness of lemon balm extracts at various concentrations (1, 2.5, 5, 7.5, and 10%) in comparison to the control (PDA without extract) was tested. The results showed that all the extract concentrations under study exerted an inhibitory activity on *Fusarium proliferatum* and *Fusarium culmorum* at different levels than the control (Figure 1 and Table 1). 

Although lemon balm extracts had a definite inhibitory effect on mycelial growth, the susceptibility of *F. culmorum* and *F. proliferatum* was different. As shown in (Table 1), 7.5 and 10% of lemon balm extract inhibited the development of *F. proliferatum*, whereas only 10% of lemon balm extract completely inhibited the growth of *F. culmorum*. In addition, other concentrations have shown an inhibitory effect on mycelium growth compared to the control. However, the results were inconsistent. For *F. proliferatum,* the inhibition percent ranged from 4.6 to 100%, whereas for *F. culmorum*, the inhibition percent ranged from 0.7 to 100%. According to the analysis of variance, the inhibitory impact of lemon balm extracts on fungal growth between the selected *Fusarium* strains was not significantly different (*p* = 0.396). 

### 2.2. The Effect of Lemon Balm Extract on ERG Content 

The effect of lemon balm extract at different concentration levels on the *F. proliferatum* and *F. culmorum* growth was analyzed by determining ERG concentration using the HPLC/PDA technique. In both strains examined, lemon balm extracts considerably decreased the ERG content compared to the control (Table 2). The ERG concentration was determined in the range between 2.9 to 141.4 and 0.7 to 45.6 µg/g for *F. proliferatum* and *F. culmorum*, respectively. The reduction percentage of ERG in treated PDA with lemon balm extract resulted in the range between 19.1 to 98.4% for *F. proliferatum* and 18.0 to 98.8% for *F. culmorum*, depending on the lemon balm concentration level. ERG concentration was significantly reduced in all tested extracts compared to the control. Furthermore, the results revealed that the extraction conditions were not statistically different. However, the ERG concentration compared to the two different strains is significantly different (Tukey HSD test, significant at *p*< 0.01). 

### 2.3. Mycotoxins Identified and Quantified by UHPLC-HESI-MS/MS and Their Reduction by Lemon Balm Extracts

The multi-mycotoxins method was used to analyze the effect of lemon balm extracts on mycotoxins production in this study (Table 3 and Table 4). Ten different mycotoxins out of 22 examined were identified. The results showed that lemon balm extract significantly reduced mycotoxins production in both *Fusarium* strains compared to the control (PDA without extract).

**Figure 2:**
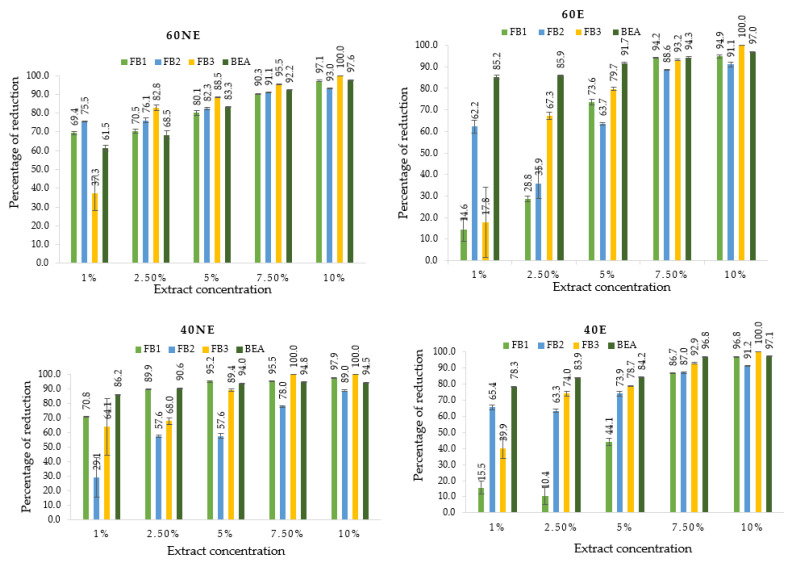
Degree of mycotoxins reduction [in %] produced by *F. proliferatum* at different concentrations of lemon balm and extraction conditions. The presented results are means ± standard deviation. Abbreviation meaning: 60NE = lemon balm extracted at 60 °C without evaporation, 40NE = lemon balm extracted at 40 °C without evaporation, 40E = lemon balm extracted at 40 °C with evaporation and 60E = lemon balm extracted at 60 °C with evaporation.

**Figure 3:**
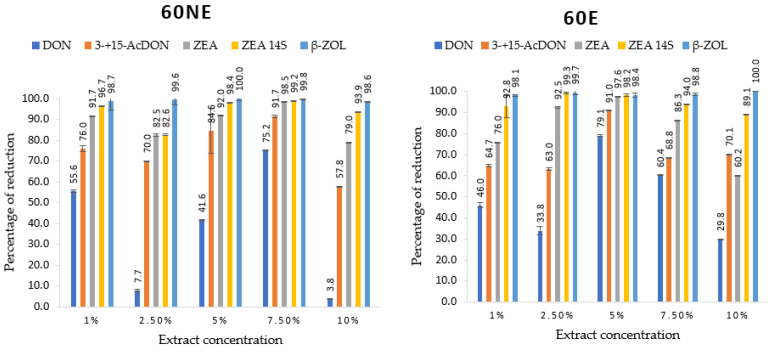

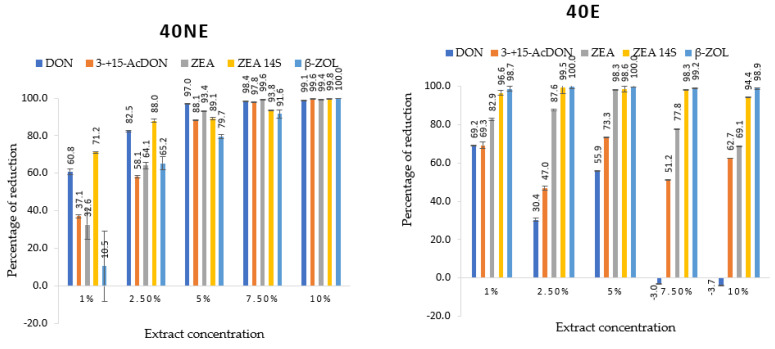
Degree of mycotoxins reduction produced by *F. culmorum* at different concentrations of lemon balm and extraction conditions. Presented results are mean ± standard deviation. Abbreviation meaning: 60NE = lemon balm extracted at 60 °C without evaporation, 40NE = lemon balm extracted at 40 °C without evaporation, 40E = lemon balm extracted at 40 °C with evaporation and 60E = lemon balm extracted at 60 °C with evaporation.

The identification and quantification of mycotoxins using LC-Q Exactive Focus Orbitrap MS showed that the *F. proliferatum* strain produced fumonisins B (FB_1_, FB_2_, and FB_3_), as well as beauvercin (BEA). BEA in the range of (0.9–13.4 µg/g) was predominant, followed by fumonisin B_1_ (FB_1_), and FB_3_ (0.00–0.05 µg/g) was the least abundant (Table 3). In general, adding lemon balm extracts to the media resulted in a significant reduction of the identified toxins compared to the control. The degree of mycotoxins reduction in all tested lemon balm extracts at different concentrations ranged between 10.4–97.1% for FB_1_, 29.1–93.0% for FB_2_, 17.8–100.0% for FB_3_, and 61.5–97.6% for BEA. These reductions depended on the concentration of extracts and the type of mycotoxin reduced (Figure 2). Statistically, the influence of lemon balm extracts on mycotoxins produced by *F. proliferatum* was significant at *p* < 0.01. However, the effect of variants (60NE, 40NE, 60E, and 40E) on the reduction of mycotoxins showed that FB_1_, FB_2_, and BEA were significant at *p* < 0.01, while for FB_3,_ all tested parameters were not significant. It is worth mentioning that the concentrations were inconsistently significant, but 7.5 and 10% were more effective compared to 1, 2.5, and 5% in all mycotoxins produced by *F. proliferatum* (Figure 3).

*F. culmorum* strain produced different mycotoxins, including trichothecenes (DON, 3-acetyldeoxynivalenol (3-AcDON), and 15-acetyldeoxynivalenol (15-AcDON)), ZEN and its derivatives (zearalenone-14-sulfate (ZEN-14S) and β-zearalenone (β-ZOL)). ZEN-14S was the most produced mycotoxin, followed by ZEN, and DON was the least produced (Table 4). The efficacy of lemon balm extract at different concentrations in mycotoxins’ reduction was as follows: DON was reduced in the range 46.0–99.1%, 3- + 15-AcDON (7.7–100%), ZEN (32.6–99.7%), ZEN-14S (−3.2–99.8%) and β-ZOL (−3.1–100%). Significant differences were observed compared to the control in all tested concentrations of lemon balm extracts (Table 4 and Figure 3). Extraction parameters (60NE, 40NE, 60E, and 40E) did not alter the reduction of DON, 3- + 15-AcDON, ZEN, and β-ZOL significantly at *p* < 0.01, while all parameters were significant for ZEN-14S. Different concentrations were significantly inconsistent as the extraction conditions didn’t differ between them. Consequently, 5, 7.5, and 10% were the most effective concentrations to reduce mycotoxins produced by the *F. culmorum* strain used in this study.

## 3. Discussion

Very few reports are available on the control of *Fusarium* spp. and their mycotoxins using plant extracts. Therefore, the goal of this study was to evaluate the antifungal efficacy of lemon balm extracts obtained by SC-CO_2_ at different concentrations in reducing the growth and mycotoxins biosynthesis of *Fusarium proliferatum* and *Fusarium culmorum*. Lemon balm is a valuable medicinal herb with a long history of usage in traditional medicine [53]. Its applications in pharmacology, phytopathology, and food preservation have also been discovered [54]. The general antimicrobial activity of lemon balm extracts has been reported [42,45]. However, its application as a natural antifungal agent in the agricultural field was not well exploited. The work presented here takes a different approach to estimate the influence of lemon balm extracts on fungal growth in vitro: a food poisoned approach on a Petri dish, chromatographic analyses of ERG on PDA, and estimation of the level of different mycotoxins reduction using the LC-Q Exactive Focus Orbitrap MS method. The inhibitory effect of the extract increases when the concentration of extracts increases. This agrees with the findings of Sibel et al. [44], who found that sage and lemon balm essential oils had an inhibitory effect on the mycelium growth of ochratoxigenic *Penicillium verrucosum* [44]. Gakuubi et al. [28] also showed that essential oils of *Eucalyptus camaldulensis* exhibited a positive correlation between concentration and activity against tested *Fusarium* spp., and at a concentration of 10µL/mL, all tested fungi were completely inhibited [28]. Aziz & Al-Askar [55] studied the antifungal activity of plant extracts (seeds and rinds of camel thorn, caper roots, and pomegranate rinds) against the linear growth of five fungi at different concentrations (3, 6, and 9%) and showed that all tested plant extracts had varying antifungal activity against *R. solani*. There was an inverse relationship between the concentration of the plant extracts and the linear growth of *R. solani,* i.e., as the concentration increases, the linear growth decreases [55]. Manssouri et al. [56] used poisoned food (PF) and volatile activity (VA) methods to test the antifungal activity of EO from *Ammodaucus leucotrichus* fruits against three phytopathogenic fungi that cause apple deterioration, including *Botrytis cinerea*, *Penicillium expansum*, and *Rhizopus stolonifer*, demonstrating the EO considerable antifungal activity against the studied phytopathogenic fungi (*p* < 0.05). In the PF approach, the MICs (minimum inhibitory concentrations) for *B. cinerea* and *P. expansum* were 0.5 L/mL and 1 L/mL for *R. stolonifer*. In the VA experiment, *B. cinerea* and *P. expansum* mycelial growth were inhibited entirely at MIC = 0.125 L/mL air, whereas *R. stolonifer* mycelial growth was completely inhibited at MIC = 0.25 L/mL air [56]. Another study, however, has found the contrary effect of plant extract. They investigated the antifungal activity of the aqueous phase of lemon balm extracts against different fungal species (*Alternaria alternata*, *Fusarium oxysporum*, *Aspergillus flavus,* and *Beauveria bassiana*) by measuring the mycelium diameter and reported that lemon balm extracts enhanced fungal growth, while total inhibition of aflatoxins production was observed [36].

The inhibition of *F. proliferatum* and *F. culmorum* growth by tested lemon balm extracts obtained using SC-CO_2_ was confirmed by HPLC analysis of ERG content and UHPLC- HESI-MS/MS for mycotoxins determination. ERG (an indicator of fungal biomass) is a sterol molecule found in the membrane of fungi that is important for their proliferation. It helps maintain membrane fluidity and integrity [57,58]. ERG content depends on the physiological state and general growth conditions [59]. As a result, its examination is useful in determining if a fungus cell is alive or dead [58]. The addition of lemon balm extracts generally resulted in a considerable reduction of ERG content and mycotoxins biosynthesis in both fungi studied, which is in accordance with previous reports on antifungal effects of plant extracts or essential oils against fungi. Hu et al. [58] found that natural EO derived from turmeric (*Curcuma longa* L.) had significant antifungal effects on mycelial growth, spore germination, and aflatoxin production by *Aspergillus flavus* in a dose-dependent manner, as well as inhibiting ERG synthesis [58]. Furthermore, several studies have shown that the composition of extracts or essential oils significantly influences their antifungal properties. In this work, the analysis of the main component of lemon balm extract was not studied, but citral (neral and geranial), citronellal, and E-caryophyllene, as well as isomenthone, rosmarinic acid, were previously identified as antimicrobial components of lemon balm [42]. De Lira Mota et al. [57], while evaluating the antifungal activity of the EO of *Thymus vulgaris* and its constituents (thymol and p-cymene) against *Rhizopus oryzae* using microbiological screening, determination of minimal inhibitory concentration (MICs), and minimal fungicidal concentration (MFCs), and measuring the effects on mycelial growth and germination of sporangiospores and interaction with ERG found considerable suppression of mycelium development and germination due to EOs and thymol. MIC of EO and thymol ranged from 128 to 512 μg/mL, whereas the MFC of EO and thymol varied from 512 to 1024 and 128 to 1024 μg/mL, respectively [57]. Ginger essential oil (GEO; *Zingiber officinale* Roscoe) was tested for antifungal efficacy against *Fusarium verticillioides* by Yamamoto-Riberio et al. [60]. GEO had inhibitory activity, with a MIC of 2500 μg/mL, and at 4000 and 5000 μg/mL, it reduced ERG production by 57% and 100%, respectively. At GEO concentrations of 4000 and 2000 μg/mL, the inhibitory effect on fumonisin B_1_ and B_2_ (FB_1–2_) production was considerable. Thus, the concentration of GEO affected the suppression of fungal biomass and fumonisin synthesis [60].

Although ERG is an excellent predictor of fungal development, it is not a reliable indicator of mycotoxins contamination since not all fungi synthesize mycotoxins, and mycotoxins remain present when the fungus is not viable. In the present work, a multi-mycotoxins method (UHPLC-HESI-MS/MS) was used as an advanced, reliable, and sensitive method that helps detect multiple mycotoxins in a single analysis. A reduction of the produced mycotoxins (fumonisins-FBs, DON, 3- + 15-AcDON, ZEN, and its derivatives) was observed in the presence of lemon balm extract compared to the control. BEA was highly produced but also sensitive to lemon balm extract as it was the one that has been highly reduced compared to FBs (Figure 2). BEA is biosynthesized as a secondary metabolite by numerous toxigenic fungi, primarily of the *Fusarium* genus but also of other species such as *Beauveria bassiana* [61]. Our results agree with previous studies that reported antifungal properties of plant extracts against *Fusarium* species. Barral et al. [62] showed that *F. proliferatum* produced higher levels of BEA compared to FBs, and the phenolic extract was highly effective in reducing FBs and BEA [62]. Velluti et al. [13] studied the inhibitory effect of cinnamon, clove, oregano, palmarosa, and lemongrass oils on growth and FB_1_ production by three isolates of *F. proliferatum* in irradiated maize grain at 0.995 and 0.950 aw and at 20 °C and 30 °C. All EOs tested inhibited the growth of *F. proliferatum* FB_1_ biosynthesis [13]. Sumalan et al. [63] reported the inhibitory potential of *Fusarium* species and the reduction of fumonisins and DON contents on wheat kernels using essential oils [63].

*F. culmorum* produced DON, 3-AcDON,15-AcDON, ZEN, ZEN-14S, and β-ZOL. The reduction effects of lemon balm extract were significant compared to the control. Results showed that ZEA-14S was highly produced (1.2–266.2 µg/g), and β-ZOL was the least produced (0.0–0.5 µg/g). These mycotoxins produced by *F. culmorum* are categorized into two main groups: B trichothecenes (DON, 3-AcDON + 15-AcDON) and ZEN with its modified forms (ZEN, ZEN-14S, and β-ZOL). The modified forms of mycotoxins can originate from the metabolic pathways of the infected plant or may be synthesized by fungi [64]. Modified mycotoxins can coexist alongside free mycotoxin, as shown in this study, and in some situations, the concentration of modified mycotoxins may exceed the amount of free mycotoxin. In addition, there is evidence that some modified mycotoxins can be transformed back into the original mycotoxin during digestion in people and animals, potentially causing health problems [65]. In this study, all those modified forms were of fungal origin, and the correlation between ZEN and ZEN-14S contents was observed. Perczak et al. [66] investigated the antifungal activity of essential oils against the growth of *Fusarium graminearum* and *F. culmorum*. They discovered that the selected EOs significantly inhibited the growth of tested *Fusarium* species (90.9–99.9%), as determined by ERG quantification, and that EO application resulted in a significant reduction of ZEN (99.1–99.9%) and group B trichothecenes (94.5–100%) was observed [66]. 

## 4. Conclusions

In conclusion, the current investigation found potent antifungal properties against *F*. *proliferatum* and *F. culmorum*. All concentrations of SC-CO_2_-extracted lemon balm have shown a suppressive effect on ERG content and mycotoxins biosynthesis compared to the control (PDA without extract). The lemon balm concentrations of 5, 7.5, and 10% were most effective in inhibiting *F. proliferatum*, whereas 7.5 and 10% were most efficient in inhibiting *F. culmorum*. Lemon balm extract has shown a concentration-dependent inhibitory effect on mycelium growth, which means the higher the lemon balm concentration, the stronger the inhibition of mycelium development. However, the effect was inconsistent for mycotoxins biosynthesis, where lowering effects depended not only on the lemon balm concentration but also on the type of mycotoxin. However, 7.5 and 10% exhibited the best overall reduction degree. In general, this investigation found that lemon balm extracts had a biocontrol effect on the selected *Fusarium* strains. To consider it as a natural alternative to synthetic fungicides against *Fusarium* species, the primary composition of the SC-CO_2_-extracted lemon balm should be revealed and its in vivo efficacy as an antifungal agent compared to commercially available antifungal agents needs to be verified.

## 5. Materials and Methods

### 5.1. Plant Material 

The plant material (Melisa officinalis, cultivar Aurea) was obtained from Warmia and Mazury certified organic farms in Poland, where no artificial fertilizers or chemical pesticides are used.

### 5.2. Fungal Material 

*Fusarium culmorum* (strain KF 191) and *Fusarium proliferatum* (strain PEA1) were isolated from wheat kernels and pea seeds, respectively. They were identified by molecular techniques [67,68] and preserved at the Institute of Plant Genetics, Polish Academy of Sciences, Poznan, Poland. 

### 5.3. Chemicals

Carbon dioxide (CO_2_, SFE grade), contained in a dip tube cylinder, was purchased from Air Products Sp, Poland. Acetonitrile (AcN), methanol (MeOH), and water for LC-MS grade were acquired from POCh (Gliwice, Poland). Formic acid and ammonium formate were obtained from Fluka (Milan, Italy). Potato dextrose agar (PDA) was supplied by Oxoid, Basingstoke, UK. All chemicals were of analytical grade.

Analytical standards purchased in ready-to-use solutions from Romer Labs (Tulln, Austria) included: ERG, FB_1_, FB_2_, FB_3_, ZEN, DON, 15-AcDON, 3-AcDON, and BEA, which was 100 µg/mL^.^ The β-ZOL concentration was 10 µg/mL. ZEN-14S (100 µg/mL) was purchased in Aokin (Berlin, Germany). Depending on solubility, the standards were dissolved in acetonitrile. All standards were stored in amber glass vials maintained at approximately minus 20 °C. A mixture of all standards necessary for a particular analytical run was prepared immediately prior to the analysis.

### 5.4. Supercritical Carbon Dioxide (SC-CO_2_) Extraction

The extraction was performed on a laboratory scale utilizing the MV-10ASFE (Waters, Manchester, MA, USA), which included a CO_2_ cylinder and a cooling system, fluid delivery module, column oven, back pressure regulator, heat exchanger, and fraction collection module, as well as ChromScope v1.20 software (Waters, Manchester, MA, USA). Five grams of lemon balm were placed in a 25 mL extraction vessel and into an oven set at the proper temperatures (40 and 60 °C) and pressure (250 bar). The CO_2_ flow rate was set at 4 mL/min, and the methanol flow rate was set at 1 mL/min. After turning on the injection valve and the backpressure regulator, the temperature and pressure were established. Each experimental run lasted 180 min, with the first dynamic time of 45 min, the static time of 15 min, and the second dynamic duration of 120 min. The obtained lemon balm extracts were divided into two parts; one part was collected in small vials and directly kept at −20 °C before further research; samples named 60NE and 40NE and the second part of extracts were subjected to evaporation using a rotary evaporator operated at 40 °C to concentrate the extracts, and the dry residues were reconstituted with pure methanol and kept in vials at −20 °C before further experiment; sample named 60E and 40E. 

### 5.5. The Effect of Lemon Balm Extracts Prepared at Different Concentration Levels on Mycelia Growth 

The antifungal activity of lemon balm extract was estimated using the method described by Salhi et al. [69] with minor modifications. The autoclaved PDA medium was mixed with prepared lemon balm extracts at different concentration levels of 1, 2.5, 5, 7.5, and 10% to have the final volume of 15 mL on each Petri dish. The homogenized mixture was poured into sterilized Petri dishes and left for a few minutes to solidify. After that, the center of each Petri dish was inoculated with a 6 mm diameter disc of fungal mycelium, taken from pure culture (7 days old). Plates containing mycelium disc without lemon balm extracts were used as controls. All inoculated plates were incubated at 25 °C in the dark for 10 days, and the radial mycelial growth was measured each day. For each treatment, three replicates were maintained, and the experiment was repeated three times. Finally, the antifungal activity of each extract was calculated in terms of the inhibition percentage of mycelia growth by using the following formula:

The inhibition of mycelium growth (%) = (C − T)/C × 100 (1)

where C is the average diameter of fungal growth on control Petri dishes, and T is the average diameter of fungal growth on Petri dishes with lemon balm extract.

After the incubation period, the whole PDA with mycelia was freeze-dried, milled, and prepared for chromatographic analysis. 

### 5.6. Determination of ERG in Mycelia Grown on PDA with Lemon Balm Extract 

One hundred milligrams of dried PDA with mycelia sample were placed into test tubes and suspended in 2 mL of methanol with 0.5 mL of 2 M aqueous sodium hydroxide. Then, tightly sealed test tubes were placed inside the microwave oven (Model AVM 401/1WH, Whirlpool, Sweden), operating at 2450 MHz and 900 W maximum output. Samples were irradiated (370 W) 3 times for 10 s. After 15 min, the contents of the test tubes were neutralized with 1 mL of 1 M aqueous hydrochloric acid, and 2 mL MeOH was added. For extraction, 3 × 4 mL of *n*-pentane was added to the solutions, and the supernatants were collected into the vial and evaporated to dryness in a nitrogen stream. Prior to analysis, the dried vial with samples was dissolved in 1 mL of MeOH LC/MS grade, filtered through 15 mm syringe filters with 0.20 µm pore diameter (Chromafil, Macherey-Nagel, Duren, Germany), and 30 µL was analyzed by HPLC technique. Separation was achieved on 150 × 3.9 mm Nova Pak C-18, 4 µm column, and eluted with methanol/acetonitrile (90:10, *v*/*v*) at a flow rate of 0.6 mL/min. ERG was detected with a Waters 2996 Photodiode Array Detector (Waters Division of Millipore, Milford, MA, USA) set at 282 nm. The quantification of ERG was made by comparing peak areas with those of ERG external standards. Confirmation of ERG was achieved by comparing retention times with the external standard and by co-injection of every sixth sample with an ERG standard.

### 5.7. Analysis of Mycotoxins Using UHPLC-HESI-MS/MS

Mycotoxins produced by selected *Fusarium* strains were extracted and simultaneously determined using liquid chromatography-Q-Exactive Orbitrap mass spectrometry operating with a heated electrospray interface (UHPLC-HESI-MS/MS) (Thermo Fisher Scientific, Waltham, MA, USA). Firstly, mycotoxins extraction was done by homogenizing the mixture of 0.5 g of dried PDA with mycelia and 10 mL of acetonitrile/water solution (84:16) with 0.1% formic acid for 2 min. The entire mixture was centrifuged for 10 min at 10,730× *g*. The extract was filtered. 2 mL of the extract was evaporated in a rotary evaporator and dissolved in 600 µL methanol, sonicated for 2 min, and supplemented with 400 µL water with 0,25% formic acid. The samples were filtered by a disc filter of 0.2 µm. Secondly, mycotoxins detection and determination were analyzed with UHPLC-HESI-MS/MS. The analytes were separated on a non-porous C18 Cortecs chromatographic column (100 mm × 2.1 mm × 1.6 μm, Waters) with an inline filter located in front of the chromatographic column. The mobile phase consisted of water–methanol 90:10 (A) and methanol–water 90:10 (B); both phases had 5 mM ammonium formate and 0.2% formic acid. The following flow gradient (A/B ratio) was applied: 100:0 for 0–2 min; 75:25 for 2–3 min; 40:60 for 3–6 min; 0:100 for 6–20 min; 0:100 for 20–26 min; 100:0 for 26–28 min; 100:0 for 28–30. The flow rate was 0.3 mL/min, and sample volume (2 μL) was injected into the system. Lastly, the mass spectrometer was operated in switching polarity, with two scanning types: full MS data with a resolution of 70,000 and a scan range of 100 to 1000 *m*/*z* and all ion fragmentation scan (AIF) with a resolution of 17,500 and scan range 80 to 1000 *m*/*z* (Table 5). Parameters of the ion source were as follows: spray voltage 3.2 kV (for positive polarity) and 2.2 kV (for negative polarity), vaporizer temperature 350 °C, sheath gas pressure 40 arbitrary units, auxiliary gas pressure 10 arbitrary units, capillary temperature 250 °C. 

### 5.8. Statistical Analysis

Experimental data were statistically evaluated using the Statgraphics 4.1 software package (Graphics Software System, STCC, Inc., Rockville, MD, USA). Conventional statistics were used to calculate the means and standard deviations. A one-way ANOVA was used to assess the significance of the differences between the determined ERG and mycotoxin concentrations in PDA mycelium. Post-hoc Tukey HSD test at α = 0.01 was used for the paired tests.

## Figures and Tables

**Table 1:** Effect of the lemon balm extracts (1.0; 2.5; 5.0; 7.5 and 10% concentration) on the mycelium growth for *F. proliferatum* and *F. culmorum*.

Variants	Extract Concentrations (%)	Mycelium Growth Inhibition (%)	
		*F. proliferatum*	*F. culmorum*
60NE	1	13.0 ± 0.1 ^ab^	2.5 ± 0.0 ^ab^
	2.5	35.0 ± 1.8 ^abc^	30.7 ± 1.9 ^abcd^
	5	86.7 ± 0.1 ^de^	48.2 ± 2.1 ^bcde^
	7.5	96.1 ± 0.2 ^e^	100.0 ± 0.0 ^f^
	10	100.0 ± 0.0 ^e^	100.0 ± 0.0 ^f^
40NE	1	7.4 ± 0.0 ^ab^	4.1 ± 0.9 ^ab^
	2.5	48.9 ± 0.2 ^bcd^	22.3 ± 1.5 ^abc^
	5	71.9 ± 0.5 ^cde^	59.7 ± 1.1 ^cdef^
	7.5	100.0 ± 0.0 ^e^	96.1 ± 0.5 ^ef^
	10	100.0 ± 0.0 ^e^	100.0 ± 0.0 ^f^
40E	1	9.4 ± 0.0 ^ab^	1.7 ± 1.3 ^a^
	2.5	24.0 ± 0.2 ^ab^	17.2 ± 1.0 ^abc^
	5	72.7 ± 1.2 ^cde^	90.0 ± 0.4 ^ef^
	7.5	100.0 ± 0.0 ^e^	96.8 ± 0.3 ^ef^
	10	100.0 ± 0.0 ^e^	100.0 ± 0.0 ^f^
60E	1	4.6 ± 0.0 ^ab^	0.7 ± 1.8 ^a^
	2.5	33.4 ± 0.6 ^abc^	54.5 ± 0.4 ^cdef^
	5	81.9 ± 0.5 ^cde^	80.5 ± 0.1 ^def^
	7.5	100.0 ± 0.0 ^e^	94.3 ± 0.2 ^ef^
	10	100.0 ± 0.0 ^e^	100.0 ± 0.0 ^f^

All values are means of three replicates ± standard deviation. The superscript of different letters in rows are significantly different (Tukey’s HSD test, significant at *p* < 0.01). Abbreviation meaning: 60NE = lemon balm extracted at 60 °C without evaporation, 40NE = lemon balm extracted at 40 °C without evaporation, 40E = lemon balm extracted at 40 °C with evaporation and 60E = lemon balm extracted at 60 °C with evaporation.

**Table 2:** Ergosterol (ERG) content [µg/g] and percentage of its reduction [%] in PDA treated with different concentrations of lemon balm extracts (1, 2.5, 5, 7.5, and 10%) after inoculation with *Fusarium* species.

Variants	Extract Concentrations [%]	ERG Concentration [µg/g] and Percentage of Reduction [%]			
		*F. proliferatum*		*F. culmorum*	
		[µg/g]	[%]	[µg/g]	[%]
Control (without extract)		178.0 ± 23.4 ^g^	-	55.7 ± 5.3 ^i^	-
60NE	1	74.3 ± 6.4 ^e^	58.3	28.5 ± 3.9 ^fg^	48.8
	2.5	31.7 ± 3.8 ^bcd^	82.2	14.2 ± 3.0 ^bcd^	74.4
	5	23.7 ± 3.9 ^abcd^	86.7	8.6 ± 0.4 ^abcd^	84.6
	7.5	21.09 ± 2.0 ^abc^	88.2	7.5 ± 0.8 ^abc^	86.6
	10	12.2 ± 3.3 ^abc^	93.2	7.6 ± 0.7 ^abcd^	86.3
40NE	1	141.4 ± 9.5 ^f^	20.6	45.6 ± 4.4 ^h^	18.0
	2.5	47.6 ± 8.8 ^d^	73.3	28.7 ± 2.8 ^fg^	48.5
	5	35.3 ± 4.8 ^cd^	80.2	26.5 ± 1.4 ^ef^	52.4
	7.5	15.3 ± 4.3 ^abc^	91.4	16.1 ± 2.5 ^cd^	71.1
	10	6.7 ± 0.7 ^a^	96.2	0.7 ± 0.1 ^a^	98.8
40E	1	144.0 ± 6.8 ^f^	19.1	36.5 ± 5.0 ^gh^	34.4
	2.5	26.3 ± 5.4 ^abcd^	85.2	27.9 ± 3.3 ^fg^	49.9
	5	21.1 ± 3.3 ^abc^	88.2	6.8 ± 0.7 ^abc^	87.8
	7.5	13.0 ± 3.4 ^abc^	92.7	4.8 ± 0.3 ^ab^	91.4
	10	8.9 ± 1.6 ^ab^	95.0	3.4 ± 0.4 ^a^	94.0
60E	1	47.7 ± 5.1 ^d^	73.2	33.5 ± 3.6 ^fg^	39.8
	2.5	32.8 ± 3.4 ^bcd^	81.6	17.3 ± 4.2 ^de^	68.9
	5	11.7 ± 2.3 ^abc^	93.4	6.41 ± 1.0 ^abc^	88.5
	7.5	9.4 ± 2.0 ^ab^	94.7	3.5 ± 0.6 ^a^	93.7
	10	2.9 ± 0.5 ^a^	98.4	2.5± 0.5 ^a^	95.5

All values are means of three replicates ± standard deviation. The superscript of different letters in rows are significantly different (Tukey’s HSD test, significant at *p* < 0.01). Abbreviation meaning: 60NE = lemon balm extracted at 60 °C without evaporation, 40NE = lemon balm extracted at 40 °C without evaporation, 40E = lemon balm extracted at 40 °C with evaporation and 60E = lemon balm extracted at 60 °C with evaporation.

**Table 3:** The effect of different concentrations of lemon balm extracts (1, 2.5, 5, 7.5, and 10%) on *F. proliferatum* mycotoxins after 10 days of incubation at 25 °C on a PDA medium.

Variants	Extract Concentrations (%)	Mycotoxins (µg/g)			
		FB_1_	FB_2_	FB_3_	BEA
Control (without extract)		4.3 ± 0.1 ^j^	0.4 ± 0.1 ^h^	0.2 ± 0.0 ^f^	36.4 ± 0.6 ^l^
60NE	1	1.3 ± 0.0 ^f^	0.1 ± 0.0 ^bcde^	0.1 ± 0.0 ^e^	13.4 ± 0.6 ^k^
	2.5	1.3 ± 0.0 ^f^	0.1 ± 0.0 ^bcde^	0.03 ± 0.0 ^abcd^	11.2 ± 0.8 ^j^
	5	0.8 ± 0.1 ^de^	0.1 ± 0.0 ^bcde^	0.02 ± 0.0 ^abc^	6.1 ± 0.8 ^h^
	7.5	0.4 ± 0.0 ^bc^	0.03 ± 0.0 ^ab^	0.01 ± 0.0 ^ab^	2.8 ± 0.1 ^def^
	10	0.1 ± 0.0 ^a^	0.02 ± 0.0 ^a^	n.d.^a^	0.9 ± 0.0 ^a^
40NE	1	1.2 ± 0.0 ^f^	0.3 ± 0.0 ^g^	0.1 ± 0.0 ^e^	4.9 ± 0.1 ^g^
	2.5	0.4 ± 0.0 ^bc^	0.2 ± 0.0 ^f^	0.1 ± 0.0 ^e^	3.5 ± 0.1 ^f^
	5	0.2 ± 0.0 ^ab^	0.2 ± 0.0 ^f^	0.02 ± 0.0 ^abc^	2.2 ± 0.1 ^de^
	7.5	0.2 ± 0.0 ^ab^	0.1 ± 0.0 ^bcde^	n.d.^a^	1.9 ± 0.1 ^bcd^
	10	0.1 ± 0.0 ^a^	0.04 ± 0.0 ^abc^	n.d.^a^	2.0 ± 0.1 ^bcd^
40E	1	3.6 ± 0.2 ^i^	0.1 ± 0.0 ^bcde^	0.1 ± 0.0 ^e^	8.0 ± 0.1 ^i^
	2.5	3.8 ± 0.2 ^i^	0.1 ± 0.0 ^bcde fg^	0.04 ± 0.0 ^bcd^	5.8 ± 0.1 ^gh^
	5	2.4 ± 0.1 ^g^	0.1 ± 0.0 ^bcde^	0.03 ± 0.0 ^abcd^	5.8 ± 0.1 ^gh^
	7.5	0.6 ± 0.0 ^cd^	0.04 ± 0.0 ^abc^	0.01 ± 0.0 ^ab^	1.1 ± 0.1 ^ab^
	10	0.1 ± 0.0 ^a^	0.03 ± 0.0 ^ab^	n.d.^a^	1.0 ± 0.1 ^ab^
60E	1	3.6 ± 0.2 ^i^	0.1 ± 0.0 ^bcde^	0.1 ± 0.0 ^e^	5.6 ± 0.3 ^gh^
	2.5	3.1 ± 0.1 ^h^	0.2 ± 0.0 ^f^	0.05 ± 0.0 ^cd^	5.1 ± 0.1 ^g^
	5	1.1 ± 0.1 ^ef^	0.1 ± 0.0 ^bcde^	0.03 ± 0.0 ^abcd^	3.2 ± 0.2 ^ef^
	7.5	0.2 ± 0.0 ^abc^	0.04 ± 0.0 ^abc^	0.01 ± 0.0 ^ab^	2.2 ± 0.1 ^cde^
	10	0.2 ± 0.0 ^abc^	0.03 ± 0.0 ^ab^	n.d.^a^	1.2 ± 0.1 ^abc^

All values are means of three replicates ± standard deviation. The superscript of different letters in rows are significantly different (Tukey’s HSD test, significant at *p* < 0.01). Abbreviation meaning: 60NE = lemon balm extracted at 60 °C without evaporation, 40NE = lemon balm extracted at 40 °C without evaporation, 40E = lemon balm extracted at 40 °C with evaporation and 60E = lemon balm extracted at 60 °C with evaporation, n.d.—not detected.

**Table 4:** The effect of different concentrations of lemon balm extracts (1, 2.5, 5, 7.5, and 10%) on *F. culmorum* mycotoxins after 10 days of incubation at 25 °C on a PDA medium.

Variants	Extract Concentrations (%)	Mycotoxins [µg/g]				
		DON	3- + 15- AcDON	ZEN	ZEN-14S	β-ZOL
Control (without extract)		29.8 ± 0.5 ^j^	40.3 ± 0.7 ^h^	250.6 ± 12.1 ^k^	672.4 ± 16.7 ^h^	0.5 ± 0.0 ^f^
60NE	1	13.3 ± 0.2 ^h^	37.2 ± 0.7 ^h^	146.4 ± 0.3 ^i^	166.5 ± 1.0 ^de^	0.5 ± 0.0 ^f^
	2.5	7.2 ± 0.3 ^d^	12.1 ± 0.1 ^d^	38.6 ± 1.4 ^de^	56.1 ± 3.3 ^bc^	0.2 ± 0.0 ^d^
	5	2.5 ± 0.1 ^b^	7.0 ± 5.3 ^c^	20.2 ± 0.9 ^bcd^	9.9 ± 7.3 ^a^	0.1 ± 0.0 ^bc^
	7.5	1.0 ± 0.1 ^a^	7.0 ± 0.3 ^c^	4.1 ± 0.0 ^abc^	5.1 ± 0.1 ^a^	0.03 ± 0.0 ^ab^
	10	0.4 ± 0.0 ^a^	0.2 ± 0.0 ^a^	0.1 ± 0.0 ^a^	1.3 ± 0.1 ^a^	0.01 ± 0.0 ^a^
40NE	1	11.7 ± 0.5 ^g^	25.4 ± 0.5 ^f g^	168.9 ± 20.8 ^j^	193.9 ± 3.3 ^e^	0.4 ± 0.1 ^f^
	2.5	5.2 ± 0.2 ^c^	16.9 ± 0.4 ^e^	89.9 ± 5.0 ^g^	80.5 ± 6.0 ^bc^	0.2 ± 0.0 ^cd^
	5	0.9 ± 0.6 ^a^	4.8 ± 0.1 ^bc^	16.6 ± 0.2 ^abc^	73.5 ± 5.9 ^bc^	0.1 ± 0.0 ^bc^
	7.5	0.5 ± 0.0 ^a^	0.9 ± 0.0 ^ab^	1.1 ± 0.0 ^ab^	41.5 ± 0.9 ^ab^	0.04 ± 0.0 ^ab^
	10	0.3 ± 0.0 ^a^	0.1 ± 0.0 ^a^	1.5 ± 0.2 ^ab^	1.2 ± 0.1 ^a^	n.d. ^a^
40E	1	9.2 ± 0.1 ^e^	28.1 ± 0.8 ^g^	110.5 ± 2.1 ^h^	679.4 ± 11.7 ^h^	0.5 ± 0.0 ^f^
	2.5	9.2 ± 0.3 ^e^	21.4 ± 0.6 ^f^	67.0 ± 1.6 ^f^	328.4 ± 25.1 ^g^	0.2 ± 0.0 ^cd^
	5	5.1 ± 0.1 ^c^	5.0 ± 0.1 ^bc^	4.3 ± 0.0 ^abc^	149.4 ± 12.5 ^d^	0.1 ± 0.0 ^cd^
	7.5	1.0 ± 0.0 ^a^	0.2 ± 0.0 ^a^	3.5 ± 0.1 ^abc^	11.4 ± 0.8 ^a^	0.02 ± 0.0 ^ab^
	10	0.4 ± 0.0 ^a^	n.d. ^a^	0.1 ± 0.0 ^a^	5.6 ± 0.2 ^a^	0.01 ± 0.0 ^a^
60E	1	16.1 ± 0.5 ^i^	26.7 ± 0.3 ^g^	52.4 ± 0.2 ^ef^	266.2 ± 43.0 ^f^	0.3 ± 0.0 ^e^
	2.5	10.5 ± 0.7 ^f^	14.9 ± 0.3 ^de^	22.6 ± 1.3 ^cd^	209.9 ± 2.7 ^e^	0.1 ± 0.0 ^cd^
	5	7.2 ± 0.2 ^d^	3.0 ± 0.1 ^abc^	6.0 ± 0.0 ^abc^	92.1 ± 4.1 ^c^	0.2 ± 0.0 ^d^
	7.5	2.1 ± 0.1 ^b^	0.3 ± 0.0 ^a^	4.4 ± 0.7 ^abc^	40.7 ± 0.5 ^ab^	0.05 ± 0.0 ^ab^
	10	0.6 ± 0.0 ^a^	0.1 ± 0.0 ^a^	4.1 ± 0.1 ^abc^	8.1 ± 0.4 ^a^	n.d. ^a^

All values are means of three replicates ± standard deviation. The superscript of different letters in rows are significantly different (Tukey’s HSD test, significant at *p* < 0.01). Abbreviation meaning: 60NE = lemon balm extracted at 60 °C without evaporation, 40NE = lemon balm extracted at 40 °C without evaporation, 40E = lemon balm extracted at 40 °C with evaporation and 60E = lemon balm extracted at 60 °C with evaporation, n.d.—not detected.

**Table 5:** Ions mass and retention time for individual mycotoxins.

Analyte	Ion Mass [*m*/*z*]	Retention Time [min]	LOQ * [ng/g]	LOD * [ng/g]
FB_1_	722.837 (M + H)^+^	8.41	50	15
FB_2_	706.837 (M + H)^+^	10.56	50	15
FB_3_	706.837 (M + H)^+^	9.45	50	15
DON	297.327 (M + H)^+^	3.21	80	24
3- + 15-AcDON	339.357 (M + H)^+^	5.80	20	6
ZEN	317.352 (M − H)^−^	9.44	5	1.5
ZEN-14S	397.081 (M − H)^−^	7.24	10	3
Β-ZOL	319.372 (M − H)^−^	8.31	4	1.2
BEA	801.983 (M + NH_4_)^+^	17.86	2	0.6

* LOQ—limit of quantification; LOD—limit of detection.
